# Substance Use Disorders and Behavioral Addictions During the COVID-19 Pandemic and COVID-19-Related Restrictions

**DOI:** 10.3389/fpsyt.2021.653674

**Published:** 2021-04-16

**Authors:** Nicole M. Avena, Julia Simkus, Anne Lewandowski, Mark S. Gold, Marc N. Potenza

**Affiliations:** ^1^Department of Neuroscience, Icahn School of Medicine at Mount Sinai, New York, NY, United States; ^2^Princeton University, Department of Psychology, Princeton, NJ, United States; ^3^Columbia College, Columbia University, New York, NY, United States; ^4^Department of Psychiatry and National Council, School of Medicine, Institute for Public Health, Washington University in St Louis, Missouri, TX, United States; ^5^Department of Psychiatry and the Child Study Center, Yale School of Medicine, New Haven, CT, United States; ^6^Connecticut Council on Problem Gambling, Wethersfield, CT, United States; ^7^Connecticut Mental Health Center, New Haven, CT, United States; ^8^Department of Neuroscience, Yale University, New Haven, CT, United States

**Keywords:** COVID-19 pandemic, food addiction, gambling, mental health services, substance-related disorders, addictive behaviors

## Abstract

COVID-19 was first identified in Wuhan, China in December of 2019 and appeared in the United States 1 month later. Between the onset of the pandemic and January 13, 2021, over 92 million people have tested positive for the virus and over 1.9 million people have died globally. Virtually every country in the world has been impacted by this virus. Beginning in March 2020, many U.S. state governments enforced a “quarantine” to respond to the growing health crisis. Citizens were required to remain at home; schools, restaurants, and non-essential businesses were forced to close, and large gatherings were prohibited. Americans' lives were transformed in a span of days as daily routines were interrupted and people were shuttered indoors. Mounting fear and unpredictability coupled with widespread unemployment and social isolation escalated anxiety and impacted the mental health of millions across the globe. Most (53%) U.S. adults reported that the coronavirus outbreak has had a negative impact on their mental health, including inducing or exacerbating use of alcohol, drugs, gambling and overeating as coping mechanisms. In this paper, we will examine substance use and addictive behaviors that have been used to manage the stress and uncertainty wrought by the COVID-19 pandemic. We review the changing treatment landscape as therapy pivoted online and telemedicine became the norm.

## Introduction

COVID-19 appeared on January 15th, 2020 in the United States as a novel coronavirus about which scientists and doctors knew very little (1). In efforts to mitigate the spread of the virus and not tax healthcare resources, a “quarantine” began in March. Most state governments imposed stay-at home orders, requiring schools, restaurants, and non-essential businesses to close, forbidding large gatherings, prohibiting travel and enforcing spatial distancing. Nationwide restrictions did not start to ease until May, and as of this writing, many of these restrictions remain in place in certain regions of the country (2).

The COVID-19 pandemic and subsequent quarantine and lockdown restrictions have negatively impacted virtually every segment of the U.S. population. The healthcare system has been strained due to mounting COVID-19 cases^1^. Hospitals have suffered economic losses from reductions in elective procedures, limitations on routine medical services and the high cost of personal protective equipment (PPE) (3). Individually, people were faced seemingly overnight with fears over contracting this virus with unknown outcomes, altered life responsibilities including juggling home-schooling of children, worries about the health of their families and friends, and, in some cases, experiences of food insecurity, isolation and job loss.

It is important to note, while COVID-19 has often been referred to as a pandemic, and it is from a purely scientific standpoint, the term syndemic, coined first by anthropologist Merrill Singer in the 1990s has been used to describe this outbreak as well. The specificity of a syndemic is that it involves biological and social interactions and takes into account socioeconomic disparities that cause certain communities to be more heavily affected by the virus than others. These communities usually lack access to healthcare and tend to be low-income communities. They often have higher occurrences of comorbidities that make them more susceptible to the novel coronavirus. It is important to take this social aspect into account when tallying the effects of COVID-19 on the US population (4).

One of these tragic effects is the impact COVID-19 has had on the mental health of millions of Americans. Many individuals were already experiencing depression and anxiety “pre-pandemic,” with an estimated 9.5 percent of Americans utilizing mental health services in 2019. The pandemic likely exacerbated these conditions. Studies of the psychological impacts of quarantines during the SARS (severe acute respiratory syndrome) (2003) and Ebola (2014) epidemics demonstrated that individuals under government-imposed quarantines exhibited greater psychological distress (5), including higher levels of depression, stress, irritability, fear, exhaustion and insomnia (6). According to a study that assessed the psychological effects of quarantine measures in response to the SARS epidemic in Toronto, 31.2% of participants exhibited signs of depression and 28.9% exhibited signs of posttraumatic stress disorder. The study also showed that family and friends connected to infected individuals experienced heightened feelings of distress and depression (7). SARS was considered a serious epidemic that infected over 8,000 people worldwide and took 774 lives (8). In comparison, there have been over 22 million COVID-19 cases and over 379,000 deaths in the United States alone (9). It is also important to note, while not the main focus of this article, there has been evidence that shows that SARS-CoV2 can actually disrupt the central nervous system and create “acquired vulnerability” which can make an individual who is recovering from the virus more susceptible to developing psychiatric conditions after they have had COVID-19 (10). This is another element to consider when cataloging the impacts of COVID-19 on mental health.

When people experience increased psychological distress, they may rely on maladaptive coping mechanisms, including using alcohol and drugs, gambling and overeating. Over half of U.S. adults reported that the coronavirus outbreak has had a negative impact on their mental health. Of those adults, 12% reported an increase in alcohol or drug use (11). Gambling has also increased considerably between March and August of 2020 with Global Poker, a gambling research firm, reporting a 43% growth in the poker industry (12). Along with drugs, alcohol and gambling, Americans have turned to food to alleviate stress. A WebMD poll in May 2020 reported that 44% of women and 22% of men had already experienced weight gain just 2 months into government-imposed shutdowns. The “Quarantine 15” and #quarantineweightgain have been trending on social media since the early days of the pandemic (13).

This article will address the various ways in which the past months' quarantine has impacted the mental health of many and led to detrimental behaviors including substance, gambling and food addictions. Although others have already written about the challenges (and opportunities) emerging from these interacting phenomena (14–17), this article will add to this discussion and also address how access to treatment for mental health has changed in this new, more virtual world. The research for this publication was conducted using PubMed (Medline) and United States government resources. The keywords used to find the sources that are cited include: COVID-19, lockdown, substance use disorder, alcohol use disorder, food addiction, mental health, depression.

## Substance Use Behaviors and Disorders

Pandemic-related stress, anxiety and isolation, in addition to disrupted treatment and recovery programs, can increase the likelihood of substance misuse, addiction and relapse. Unemployment tends to contribute to increased spikes in substance abuse (18). As of May 2020, 39% of Americans lost their jobs or had their work hours curtailed due to the pandemic (19). The stress of financial uncertainty along with an increase in free time and the absence of employment repercussions can lead people to seek ongoing solace from illicit drugs. Data from the first quarter of 2020 demonstrate the effects of COVID-19 on substance abuse among Americans. From January to March of 2020, 19,146 people died from drug overdoses, compared to 16,682 people in the same quarter of 2019. The CDC estimated a record number of US drug-related deaths in 2020 (20).

A survey of 1,079 individuals with substance use disorders (SUDs) and SUD-impacted individuals was conducted by the Addiction Policy Forum (21). This study, which examined the impact of COVID-19 on individuals with SUDs, found that 74% of respondents said they had noticed changes in their emotions since the pandemic began (21). Twenty percent of respondents reported an increase in substance use, and 1% reported being impacted by experiencing a fatal overdose since the onset of the pandemic (21). Close to 5% (4.2%) of respondents reported an overdose. Other challenges that were identified included COVID-19 impacting treatment services and difficulties accessing specific services like naloxone and needle exchanges (22). The Addiction Policy Forum cited some perspectives from individuals in recovery or those with an active SUD. Some examples include: “During the last months I have felt more at risk of relapse than I ever have,” and “I have never felt true depression like I have in the past month. I know alcohol makes it worse, but I feel like I just want to make it through this time by staying comfortably numb” (21).

To make matters worse, seeking treatment for SUDs during quarantine has been extremely difficult for many. In-person treatment for opioid use disorder (OUD) and other SUDs has been offered virtually, but many who need these services do not have regular access to a computer or the internet. Unfortunately, the amount of attention healthcare providers can give to those in recovery, especially in the first few months of the pandemic, has been severely limited by the demand of attending to COVID-19 patients. PPE and hospital space are often difficult to spare for anyone not gravely ill with the virus (23).

## Alcohol Use

Amid isolation, financial difficulties and lockdowns, many have turned to alcohol to cope with anxiety and uncertainties during the pandemic. There are positive correlations between exposure to stress and alcohol and SUDs. For example, in the months following the September 11 terrorist attacks, around 30% of surveyed New York City residents reported significant increases in their consumption of cigarettes, alcohol or marijuana (24). Although bars, restaurants, and liquor stores were closed at the peak of the pandemic in March and April, studies reveal a 54% increase in national sales of alcohol during the week ending March 15, compared to this same week 1 year prior, with online alcohol sales increasing 234%. Consumers are ordering alcohol in bulk to limit their purchase frequency and buying mostly brands that they trust, increasing the number of favorable alcoholic beverages in people's households (25). While working from home, people may have access to alcohol during all hours of the day, which may contribute to drinking in the morning or during lunch breaks. One study reported that on average, alcohol was consumed 1 more day per month by 75% of adults. The frequency of alcohol consumption among adults in this study increased by 14% from 2019 to 2020 (26). Heavy-drinking episodes increased by 41% in women since the COVID-19 lockdown (27). Additionally, many states have changed their policies on carry-out purchases of liquor to help restaurants cope with the impact on restaurant business during the pandemic. According to the New York State Liquor Authority, as of March 16th 2020, businesses that sold alcoholic beverages on premise were allowed to begin selling for off-premise consumption as long as the beverages were in closed containers (28).

While many people turn to alcohol to relieve their stress and worries, the relief is typically only temporary. Instead, alcohol generally increases the symptoms of anxiety and depression, often leading to binge drinking. Those who use alcohol as a coping mechanism are more likely to develop SUDs (29). Alcohol can have serious neurological impacts, especially when used heavily and for prolonged periods of time. Alcohol interacts with several neurotransmitter receptor sites in the brain including GABA, glutamate and dopamine. Alcohol temporarily stimulates brain reward regions thus promoting drinking, but over time alcohol tends to act as a depressant (30). A common result of long-term alcohol use is the development or exacerbation of depression (31).

## Family Stress

The stress of the pandemic is taking a particular toll on parents with children at home. By the middle of March 2020, public and private elementary and secondary schools closed across the country and students were forced to transition to online learning. An August 2020 report by the U.S. Census Bureau stated that nearly 93% of households with school-age children reported some form of distance learning during the pandemic (32). Parents were often forced to facilitate online learning throughout the school day while juggling their own employment and attending to basic household needs. Over 70% of parents reported that managing distance learning for their children during the pandemic was a significant source of stress (33).

The American Psychological Association surveyed 3,000 adults between April 24 and May 4, 2020. The survey showed that the average stress level reported by parents of children under 18 was 6.7 out of 10 compared with 5.5 out of 10 for adults with no children living at home. Additionally, 46% of adults with children under 18 stated that their stress level was “high” (between 8 and 10) compared with 28% of adults without children reporting the same level of stress (33).

## Gaming and Gambling

Physical distancing, lockdowns and self-quarantines amid the coronavirus outbreak have been associated with increases in online gaming and gambling, which in turn have placed people at risk for gaming and gambling disorders. In addition, financial difficulties and unemployment may encourage gambling as people are encouraged to gamble to win money. Global Poker reported that the number of first-time online poker players increased by 255% since stay-at-home orders began (12).

College students may be particularly vulnerable to stress during the pandemic due to changes in their social lives, uncertainties regarding career prospects and shifts to online learning. In a study involving about 400 college students, 50.8% reported that their gaming had increased during the COVID-19 lockdown (34). These students acknowledged that gaming helped manage their stress related to the pandemic. General and specific practices to promote healthy gaming and internet use more generally have been suggested (35).

## Food Addiction

The term “freshman 15” is an expression that refers to the arbitrary weight that a student gains during his/her first year of college. Since the onset of the pandemic, the term “quarantine 15” has been used to refer to a 15-pound weight gain during self-isolation. Eating as a result of stress, specifically the stress during the outbreak of an infectious disease, is not uncommon among Americans (37). According to a 2013 study conducted by the American Psychological Association, 38% of adults reported overeating or eating more unhealthy foods due to stress, with 33% of these adults saying they do so because it helps distract them (36). Emotional eating tends to occur because when people are stressed, the stress hormone cortisol increases, which in turn, increases our appetite and motivations to eat (38). Eating may serve as a distraction or respite from pandemic isolation. Some highly palatable foods may trigger an addictive-like process in some individuals, activating reward-processing brain regions like drugs of abuse. Parallels exist between clinical and behavioral features of binge eating and substance use disorders (39, 40). Similar to how individuals become dependent on drugs or alcohol to manage depression and anxiety, the reliance on highly palatable foods for comfort and stress reduction may be considered as aspects of a “food addiction” (39, 41). Food addictions or eating disorders may include abnormal eating behaviors, such as excessive food intake or restriction and binging and purging, to cope with one's negative emotions. The National Eating Disorders Association reported a 78 percent increase in calls to their hotline and online chats in March and April this year compared to the same period in 2019 (42).

Among 602 Italians surveyed online between April and May 2020, almost half reported feeling anxious due to their eating habits and admitted to increasing their consumption of comfort foods to feel better. In addition, 86% of respondents reported that they felt unable to sufficiently control their diet (43). While emotional eating is not necessarily considered disordered, these habits may become problematic and unhealthy if one is routinely turning to food to manage stress and anxiety.

## How the Pandemic Has Changed the Treatment Landscape

For individuals with SUDs, the COVID-19 pandemic has resulted in changes in treatment including access to therapy, physician availability and adjustments to medication schedules. Moreover, fears associated with contracting the virus combined with rigid screening of patients resulted in a sharp decrease in psychiatric emergency room visits early in the pandemic (44). Inpatients traditionally shared bedrooms and common spaces. COVID-19 has put this system in jeopardy and strict admission criteria – including vigorous COVID-19 testing – has in part led to a reduced number of voluntary admissions to psychiatric facilities (45). Disruptions in treatment and difficulties obtaining treatment have intensified emotional distress associated with the pandemic. On March 17, 2020, the US federal government waived regulations pertaining to telemedicine and loosened restrictions to enable physicians to cross state lines for treatment (46). The last week of March saw a 154% increase in telehealth visits compared to the same period in 2019 (47). While these unprecedented changes arguably increased access to treatment for many individuals, even slight adjustments to traditional mental health care can be traumatizing and magnify the risk for an exacerbation or a recurrence of symptoms (48).

Relative to in-person treatment, online therapy may result in poorer communication and lower quality for some. Online therapy is often not ideal for people who are homeless, lack regular cell phone access or work outside of the home. Individuals in recovery may be enduring particular hardships as support group meetings such as Alcoholics Anonymous are being held virtually instead of in-person (21). Data from communication science and telemedicine group therapy show that online recovery and support services are not as beneficial as in-person services (48). A survey by the Addiction Policy Forum on 1,079 individuals with or impacted by SUDs was conducted between April 27 and May 8. The findings revealed that 34% of respondents reported changes or disruptions in their treatment or recovery support services since the onset of the COVID-19 pandemic, with 14% reporting that they have been unable to receive their needed services (21). Individuals with poly-substance use may have been particularly impacted (49). Other drawbacks of online recovery-related services include the absence of in-person activities, a lack of peer-to-peer social and emotional connections, and online distractions interfering with patients' engagement (48).

Arguably, there have been advantages to switching to online therapy. According to the American Psychological Association, online therapy can be more accessible to people living in areas where psychologists and psychiatrists are scarce (50). Teletherapy can provide more flexibility for people who previously found it difficult to visit an office, a greater sense of anonymity than in-person services, and 24/7 access to social support (48). In addition, research by Simpson and Reid (2014) discussing the therapeutic alliance in videoconference psychotherapy suggests that the relationship between therapist and patient is generally as good for telemedicine as it is for in-person therapy (48). Teletherapy may be more flexible for people who previously found it difficult to visit an office (50). A recent study found evidence that supports the importance of teletherapy by documenting the changes in mental health of a sample demographic after the beginning of the pandemic. The results from this study concluded that there was an increase in stress, fear, and other states of poor mental well-being that began after quarantine in March 2020. The fact that a survey of this type was able to be conducted in a fully virtual format bodes well for the future of telemedicine during and after the pandemic (51). In short, mental health treatment has been significantly altered by the COVID-19 pandemic, and while online therapy may present some drawbacks, new opportunities also exist.

While the COVID-19 pandemic has negatively impacted essentially every corner of the U.S. population, there is a distinctly disproportionate effect on disadvantaged, vulnerable populations. Reports from state and city health departments have revealed that Black, Latinx, and Native Americans test positive for and die of COVID-19 at a higher rate than other racial and ethnic groups. For example, while black Americans represent only 13% of the U.S. population, about 30% of all COVID-19 cases occurred in this racial group. Or, Latinx Americans, who constitute 18% of the U.S. population, accounted for 34% of total COVID-19 cases (52).

The unequal access to health care, greater dependency on low-wage or hourly paid employment, heightened psychological distress, and less access to treatment among racial minorities in the United States became undoubtedly evident this past year. There were noticeable racial and ethnic disparities in outpatient visits for substance use disorders during the surge of COVID-19. In Massachusetts, for example, a state with an early and considerable COVID-19 outbreak, outpatient visits for mental health and/or substance use disorders decreased by Hispanics (−33.0%) and non-Hispanic Blacks (−24.6%) while visits by non-Hispanic Whites increased by 10.5%. This decrease in mental health and/or substance use disorder visits among certain ethnic minority groups is likely due to lower access to employer-sponsored commercial insurance as well as a lack of access to digital technology (53).

## Conclusions

Nationwide closures and reduced mental health services have been detrimental to peoples, well-being. Many individuals will encounter repercussions from the COVID-19 pandemic for years to come. The U.S. will need to reevaluate how mental health treatment is provided during these times and when faced with future crises. The COVID-19 pandemic has demonstrated that many Americans may turn to maladaptive coping mechanisms when faced with significant disruptions to their daily lives. Future research should focus on creating adequate delivery of mental health resources and implementing strategies and methods to respond better when other crises occur (Table 1).

**Table 1:** Highlights and relevant sources.

Substance use disorders	Coping mechanisms, increased stress, predispositions: https://www.drugabuse.gov/publications/drugfacts/treatment-approaches-drug-addiction, https://www.niaaa.nih.gov/publications/brochures-and-fact-sheets/treatment-alcohol-problems-finding-and-getting-help
Disordered eating	Dealing with emotional eating, food addiction, and other forms of disordered eating: https://www.nationaleatingdisorders.org/where-do-i-start-0
Gambling	Financial struggles, willingness to take risks, boredom, online access: https://americanaddictioncenters.org/gambling-addiction
Depression	Isolation, too much worrying, world crises: https://www.psychiatry.org/patients-families/depression
Expansion of telehealth	Increased access, more flexibility, remoteness: https://www.apa.org/monitor/2017/02/online-therapy

## Author Contributions

NA: drafted outline and sections of the paper and edited paper. JS: collected research and drafted sections of the paper and edited paper. AL: collected research and drafted sections of the paper. MG: drafted and edited sections of paper. MP: drafted and edited sections of paper. All authors contributed to the article and approved the submitted version.

## Conflict of Interest

The authors declare that the research was conducted in the absence of any commercial or financial relationships that could be construed as a potential conflict of interest.

## Footnotes

^1^Worldometer. *Coronavirus Cases*. (2021). Available online at: https://www.worldometers.info/coronavirus/ (accessed January 13, 2021).
